# Unusual Presentation of Hyperandrogenism in Postmenopausal Women: A Report of Two Patients With Leydig Cell Hyperplasia

**DOI:** 10.7759/cureus.75640

**Published:** 2024-12-13

**Authors:** Frederik Duhamel, David Unuane, Stefanie Brock, Stefan Cosyns, Eric Balti

**Affiliations:** 1 Endocrine and Diabetes Unit, Universitair Ziekenhuis Brussel (UZ Brussel), Brussels, BEL; 2 Department of Internal Medicine, Vrije Universiteit Brussel (VUB), Brussels, BEL; 3 Department of Pathology, Universitair Ziekenhuis Brussel (UZ Brussel), Brussels, BEL; 4 Department of Gynaecology - Oncology, Universitair Ziekenhuis Brussel (UZ Brussel), Brussels, BEL; 5 Department of Gynaecology, Vrije Universiteit Brussel (VUB), Brussels, BEL

**Keywords:** bilateral salpingo-oophorectomy, hirsutism, hyperandrogenism, leydig cell hyperplasia, testosterone

## Abstract

Hyperandrogenism is a highly prevalent and debilitating hormonal disturbance encountered in women presenting with variable phenotypical features. Causes encompass a large spectrum of tumoral and nontumoral etiologies, depending on the patients’ age.

We report two postmenopausal patients with an unusual etiology of hyperandrogenism. Both underwent salpingo-oophorectomy. While the first patient was cured after bilateral salpingo-oophorectomy, the second patient had residual disease after unilateral surgical management. Complete disease control was achieved after the adjunction of medical treatment.

This report emphasizes the indication of bilateral salpingo-oophorectomy for the management of hyperandrogenism in postmenopausal women. A better understanding of the added value of presurgical hormonal status in further characterization of the disease phenotype is needed.

## Introduction

Androgen excess is a frequent condition in women of reproductive age. The most frequent etiology in this subpopulation is polycystic ovary syndrome [1]. However, a large spectrum of pathologies is associated with hyperandrogenism in women [2]. These include non-neoplastic and neoplastic causes with variable prevalence according to age [3].

Among non-neoplastic causes, ovarian hyperthecosis might result from an unbalanced testosterone and estrogen production due to an exacerbated decline in granulosa-mediated testosterone aromatization [3,4]. This challenging condition was first described in 2000 by Taylor et al. and its diagnostic adjudication is made better by histopathology [5,6]. A less frequent etiology of postmenopausal androgen excess is Leydig cell hyperplasia. The condition is characterized by hyperandrogenism without apparent ovarian or adrenal pathology on medical imaging [7]. The diagnosis is ascertained during histopathologic examination by visualization of widely separated nodules mainly of less than 1 cm. However, this does not always affect the overall size of the ovary [7].

Herein, we present two postmenopausal women with androgen excess due to Leydig cell hyperplasia. We discuss the diagnostic strategies and the applied therapeutic strategies as well as the resulting outcomes.

## Case presentation

Case 1

A 68-year-old patient presented with increasing libido and hirsutism. Blood work-up revealed high levels of androgens. Her medical history was positive for type 2 diabetes, arterial hypertension, and clear cell renal cell carcinoma for which she underwent right nephrectomy and ipsilateral adrenalectomy. The patient also underwent a Pfannenstiel hysterectomy for uterine fibroids.

Diagnostic Examination

**Figure 1:**
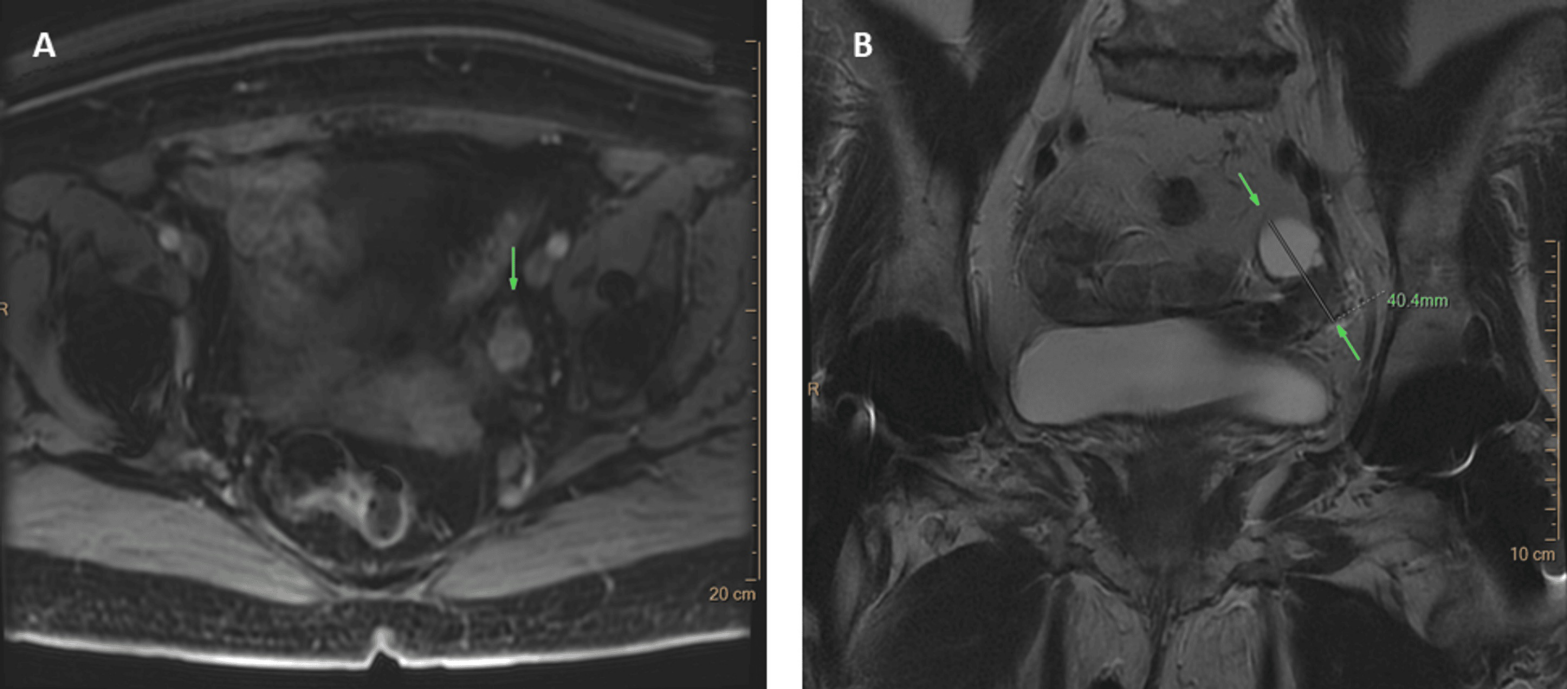
Pelvic MRI of Case 1 Left (panel A): Axial T1-weighted LAVA 90 s after IV Gd injection. The solid compartment shows enhancement (green arrow). Right (panel B): Coronal T2-weighted image. The left ovary shows a part with high signal intensity (SI) representing the cystic component (upper arrow) with adjacent microcysts and a part with low SI representing the solid compartment of the lesion (lower arrow).

On examination, the patient had normal blood pressure and heart rate. The modified Ferriman-Gallwey (mFG) score evaluating hirsutism was 13/36 (positive > 8). Also, alopecia androgenetica and clitoromegaly were observed. The results of the hormonal assessment are shown in Table 1. Additional MRI of the pelvic region showed the left cystic ovary (25 mm x 25 mm x 41 mm) without visualization of a suspected tumor (Figure 1). The right ovary could not be identified. 

**Table 1:** Hormonal analysis at presentation *Postmenopausal reference value, TSH: thyroid-stimulating hormone, ACTH: adrenocorticotropic hormone, DHEA-S: dehydroepiandrosterone sulfate, NR: not reported.

Biological parameters	Case 1	Case 2	Reference
TSH, mIU/L	3.0	1.02	0.27-4.2
Free T4, pmol/L	12.7	14.7	11.0-24.0
ACTH, ng/L	11.3	22.3	8-10h: 7.2-63
Cortisol, mcg/L	120	70.6	7-10h: 62-180
Cortisol-binding globulin, mg/L	NR	52.7	31.0-53.4
DHEA-S, mg/L	0.32	0.37	31.0-53.4
Luteinizing hormone, IU/L	29.1	36.3	1.7-8.6
Follicular-stimulating Hormone, IU/L	72.4	57	1.5-12.4
Estradiol, ng/L*	41	32.8	<50
Progesterone, mcg/L*	0.5	0.09	0.05-0.13
17-hydroxyprogesterone, mcg/L*	NR	1.42	0.3-1.7
Androstenedione, ng/L	NR	1030	330-2130
Testosterone, mcg/L	6.96	1.69	<0.12-0.41
Free testosterone, ng/L	113	30.1	60-250
Sex hormone-binding globulin, nmol/L	53	35.7	27.1-128

Treatment and Outcome

**Figure 2:**
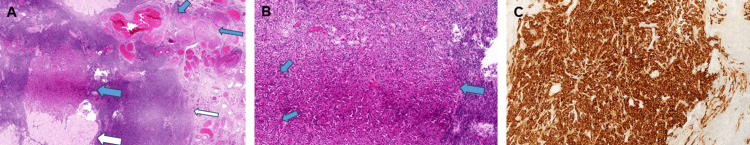
Histology findings of Case 1 Panel (A): The hilus (thin blue arrow), the stroma (thin white arrow), and corpora albicantiae (thick white arrow) in the vicinity of a Leydig cell nodule (thick blue arrow); Panel (B): The nodules of Leydig cells (left side, blue arrows) are well demarcated from the stroma on the right (arrows’ tails), Panel (C): Leydig cells are inhibin positive.

An androgen-secreting ovarian lesion was suspected, and the patient consented to bilateral salpingo-oophorectomy. However, because of multiple peritoneal adhesions most probably related to the past surgery, the left salpingo-oophorectomy could not be performed since the ovary could not be visualized despite adhesiolysis. Histologic findings of the right ovary were compatible with nodular stromal Leydig cell hyperplasia (Figure 2).

The immediate postoperative course was uneventful. Follow-up one month after surgery showed only a slight regression in the total testosterone level, but no normalization, probably related to the unilateral resection. Finasteride was initiated three months postsurgery due to persistent hirsutism. Despite a drop in dihydrotestosterone levels, there was very little improvement in hirsutism. This motivated the initiation of daily cyproterone 50 mg one year later. This resulted in normalization of testosterone levels and significant clinical improvement after a few months up to now six years of medical treatment.

Case 2

A 67-year-old patient presented with complaints of acne and a new onset of white beard hairs. Her medical history was positive for arterial hypertension, colon polyposis, diverticulitis, urge incontinence, and lumbar discectomy.

Diagnostic Examination

**Figure 3:**
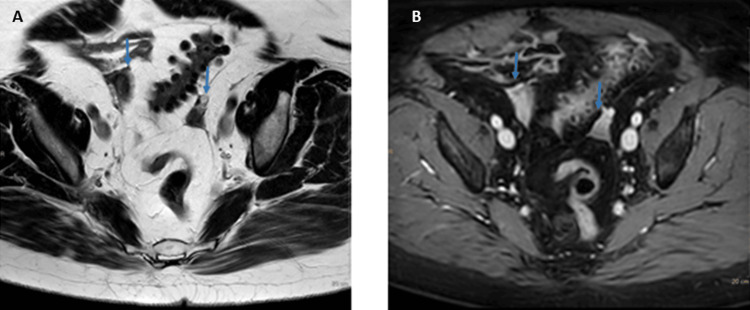
MRI showing both adnexa (blue arrows) in Case 2 Left (panel A): T2-weighted image showing the heterogeneous appearance of adnexa with the presence of a few millimetric cysts (<5 mm) and a central moderate hypointense signal intensity. Right (panel B): T1-weighted image (3d Dixon fs) after injection of Gadolinium. The solid components show enhancement.

On examination, the acne was obvious, she presented a round face, and mild buffalo hump. Blood pressure (under medical treatment for hypertension) and heart rate were normal with medical treatment. The results of her initial exploratory blood examination are shown in Table 1. Additionally, the midnight saliva cortisol and 24-hour free urine cortisol tests were normal, thus ruling out endogenous hypercortisolism. She was sent to the gynecology department for transvaginal ultrasonography which could not visualize the ovaries in this postmenopausal woman. Additional MRI identified heterogeneous cystic ovaries with normal mensuration on both sides (right ovary 24 mm x 30 mm x 28 mm and left ovary 23 mm x 27 mm x 27 mm) (Figure 3).

Treatment and Outcome

**Figure 4:**
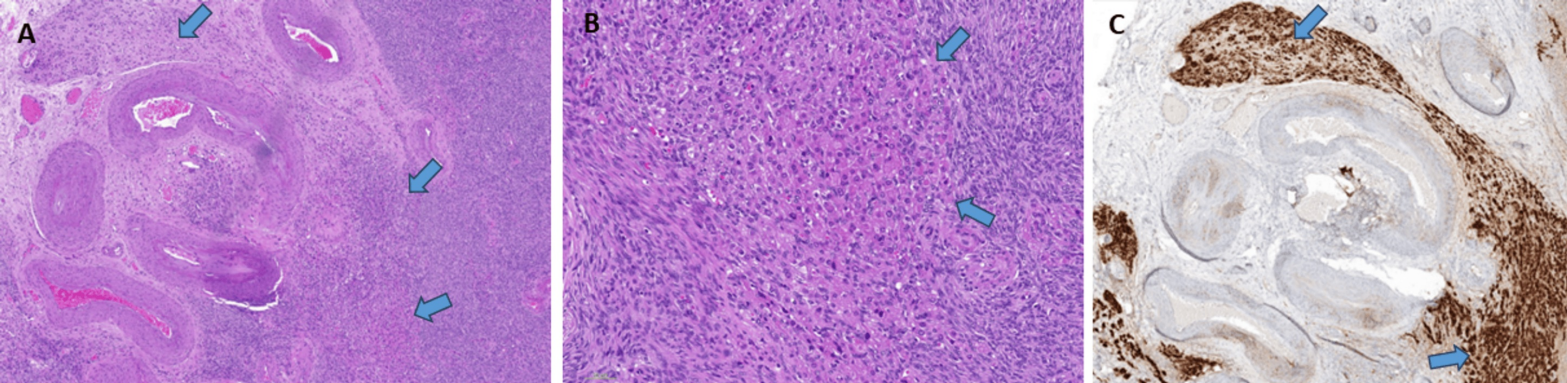
Histology findings of Case 2 Panel (A) shows the hilus with numerous large blood vessels, focally encased by a proliferation of Leydig cells (blue arrows, H&E 5x); panel (B): Leydig cells nodules are well demarcated (blue arrows) from the stroma on the right (H&E 20x); and panel (C): Leydig cells are inhibin positive (arrows, 10x).

A laparoscopic bilateral salpingo-oophorectomy was performed without any complications. The definitive histology report was compatible with bilateral Leydig cell hyperplasia (Figure 4). The patient made a full recovery and testosterone normalized soon after surgery.

## Discussion

We present cases of hyperandrogenism of ovarian origin in two postmenopausal women. In the first patient, the diagnosis was rather straightforward with a therapeutic challenge resulting from a previous surgery. Indeed, the adhesions near the left ovary hindered bilateral salpingectomy. This motivated additional pharmacological treatment after surgery with a good clinical and biological response. In the second patient, imaging suggested bilateral cystic ovaries while histology revealed Leydig cell hyperplasia. Bilateral salpingectomy resulted in optimal disease control.

Hyperandrogenism in postmenopausal women is frequently observed because of a relative physiologic rise of androgens due to a decline in estrogens and an increase in sex hormone-binding globulin [8]. Very high levels of testosterone however (> 5 nmol/L, >1.44 µg/L) should raise suspicion of an androgen-secreting tumor [3]. Imaging studies and DHEA-S determination can help differentiate between an adrenal versus ovarian source of androgen excess. An ovarian etiology, like hyperthecosis, and Leydig cell hyperplasia are the most frequent causes with a prevalence of 9.3% and 2.7%, respectively [9].

Our patients, in the sixth decade of age, presented with a relatively rapid onset of hyperandrogenism over less than one year. Their biological profiles were similar and characterized by high total testosterone levels (above 5 nmol/L), and normal DHEA-S. In the first patient, additional imaging studies following hormonal analyses were inconclusive while in the second patient, imaging suggested the presence of cystic ovaries while the definitive histology of Leydig cell hyperplasia. One of the main initial challenges in postmenopausal hyperandrogenism is discriminating ovarian from adrenal etiologies. A second challenge is the rapid identification of women bearing aggressive diseases. Besides, chronic hyperandrogenism could be associated with several metabolic morbidities which could ultimately increase mortality [10]. In a recently published observational cross-sectional study of 51 patients with postmenopausal hyperandrogenism, Luque‑Ramírez et al. reported an ovarian origin in 57% of the study population [11]. Among those, 9/29 (31%) presented with ovarian hyperthecosis and 3/29 (10.3%) with Leydig cell hyperplasia. A hint in favor of an ovarian etiology could be the higher testosterone level while acknowledging the existence of an overlapping group, with lower levels of testosterone. These individuals could further be discriminated against using DHEAS which tends to be higher in hyperandrogenism of adrenal origin. Here again, several patients with hyperandrogenism of ovarian origin had high DHEAS, and a hormonal level within the normal range was identified in about 25% of participants with an adrenal pathology [11].

In another study by Zou et al. [12], imaging was positive in 87% (27/31) of patients with confirmed virilizing ovarian tumors, but no case of Leydig cell hyperplasia out of 31 was present [12]. In this report, there seemed to be an inverse correlation between the size of ovarian tumors and age. Whether this is true for Leydig cell hyperplasia needs however to be further explored. Independently of the imaging study, surgical management (bilateral salpingo-oophorectomy) in postmenopausal women with hyperandrogenism after ruling out adrenal etiology has to be considered [7,11]. Indeed, bilateral salpingo-oophorectomy could result not only in a formal diagnosis of the underlying pathology but also in an efficient cure with quasi-immediate normalization of testosterone after surgery [13]. This was however not the case for our first patient who could only undergo unilateral oophorectomy due to the complexity of the procedure related to important peritoneal adhesions after a previous surgery. One could therefore speculate that this patient has a bilateral disease with a residual hormonal activity which was successfully managed by complementary medical treatment. This indirectly suggests the importance of systematic bilateral oophorectomy whenever surgery is considered instead of sequential salpingo-oophorectomy in postmenopausal women or premenopausal women after completion of fertility with hyperandrogenism [7,11,14].

## Conclusions

Hyperandrogenism can result in an important morbidity. Leydig cell hyperplasia is a benign condition characterized by hyperandrogenism with a challenging diagnostic ascertainment. Bilateral salpingo-oophorectomy is the best treatment modality resulting both in the diagnosis ascertainment but also in most of the cases to disease control. Whenever this is not possible, additional medical treatment could be considered. Whether or not the hormonal status could help differentiate hyperandrogenism from adrenal tumors from benign conditions needs to be further explored.
